# Niclosamide Attenuates Inflammation-Associated Profibrotic Responses in Human Subepithelial Lung Myofibroblasts

**DOI:** 10.3390/biomedicines11072032

**Published:** 2023-07-19

**Authors:** Michail Spathakis, Gesthimani Tarapatzi, Eirini Filidou, Leonidas Kandilogiannakis, Evangelos Karatzas, Paschalis Steiropoulos, Dimitrios Mikroulis, George M. Spyrou, Vangelis G. Manolopoulos, George Kolios, Konstantinos Arvanitidis

**Affiliations:** 1 Laboratory of Pharmacology, Faculty of Medicine, Democritus University of Thrace, 68100 Alexandroupolis, Greece; mspathak@med.duth.gr (M.S.); mtarapagi@gmail.com (G.T.); efilidou@hotmail.com (E.F.); leonidas.kandi@gmail.com (L.K.); emanolop@med.duth.gr (V.G.M.); gkolios@med.duth.gr (G.K.); 2 Individualised Medicine & Pharmacological Research Solutions Center (IMPReS), 68100 Alexandroupolis, Greece; 3 Institute for Fundamental Biomedical Research, BSRC “Alexander Fleming”, 16672 Vari, Greece; karatzas@fleming.gr; 4 Department of Pneumonology, Medical School, Democritus University of Thrace, 68100 Alexandroupolis, Greece; pstirop@med.duth.gr; 5 Department of Cardiothoracic Surgery, Medical School, Democritus University of Thrace, 68100 Alexandroupolis, Greece; dmikrou@med.duth.gr; 6 Bioinformatics Department, The Cyprus Institute of Neurology and Genetics, 2371 Nicosia, Cyprus; georges@cing.ac.cy

**Keywords:** niclosamide, myofibroblasts, pulmonary fibrosis, inflammation, collagen

## Abstract

Niclosamide is a commonly used helminthicidic drug for the treatment of human parasitosis by helminths. Recently, efforts have been focusing on repurposing this drug for the treatment of other diseases, such as idiopathic pulmonary fibrosis. Subepithelial lung myofibroblasts (SELMs) isolated from tissue biopsies of patients undergoing surgery for lung cancer were stimulated with TNF-α (50 ng/mL), IL-1α (5 ng/mL), added alone or in combination, and TGF-β_1_ (5 ng/mL). After treatment with niclosamide at 30 nM and 100 nM concentrations, expression of collagen type I, collagen type III, and fibronectin was studied by total RNA isolation and qRT-PCR and protein collagen secretion with the use of Sircol collagen assay. The migration of SELMs was assessed by a wound-healing assay. Niclosamide had no effect on baseline SELM fibrotic factor expression. When stimulated with TGF-β_1_, IL-1α, and/or TNF-α, SELM expression of collagen type I, type III, and fibronectin were upregulated, as was the secretion of total collagen in the culture medium. Treatment with niclosamide attenuated the effects of cytokine stimulation leading to a notable decrease in the mRNA expression of collagen type I, type III, and fibronectin in a concentration-dependent manner. SELM collagen secretion was also reduced by niclosamide at 100 nM concentration when examined at the protein level. Migration of both TGF-β_1_ stimulated and unstimulated SELMs was also inhibited by niclosamide. In this study, we highlight the anti-fibrotic properties of niclosamide on SELMs under stimulation with pro-fibrotic and pro-inflammatory cytokines, thus proposing this compound as a possible new therapeutic agent against lung fibrosis.

## 1. Introduction

Pulmonary fibrosis, defined as the abnormal accumulation and deposition of extracellular matrix in the lung, is the hallmark of several diseases affecting the lung parenchyma, collectively known as interstitial lung disease (ILD) [1]. ILD development is associated with a variety of causal factors, the most common of which are connective tissue disorders such as rheumatoid arthritis (RA) and systemic sclerosis (SSc) and exposure to environmental antigens as in the case of hypersensitivity pneumonitis [2]. Idiopathic pulmonary fibrosis (IPF) is a special case of ILD, and despite advances in the understanding of the molecular mechanisms leading to fibrosis [3], its exact pathophysiology remains to be elucidated [4]. The development of IPF has been attributed to a number of factors, including genetic variants, exposure to environmental mediators, such as cigarette smoke and infectious agents, epigenetic alterations, and cellular senescence [5]. Though rare, IPF has a major debilitating impact on a patient’s life, causing progressively worsening shortness of breath and coughing that limit the patient’s activity before ultimately reaching the point of respiratory failure and death [6].

Even though the contribution of an aberrant inflammatory response to the development of IPF remains controversial [7], there are several well-established mechanisms through which inflammatory molecules can activate fibroblasts leading to extracellular matrix (ECM) deposition in the lung [8]. It has been demonstrated that activation of a nod-like receptor (NLR) in peripheral blood mononuclear cells (PBMCs) from IPF patients leads to the induction of *TGF-β* expression [9], thus indirectly influencing the physiology of fibroblasts in IPF [10]. In addition, the pro-inflammatory cytokine TNF-α can directly induce the expression of ECM proteins in fibroblasts that promote their differentiation to a pro-fibrotic phenotype [11].

Niclosamide (5-chloro-N-(2-chloro-4-nitrophenyl)-2-hydroxybenzamide) is a well-known antiparasitic agent, most commonly used in human medicine against infestations primarily caused by cestodes [12]. As a member of the group of salicylanilides, niclosamide is believed to exert its anti-helminthic effects through the uncoupling of oxidative phosphorylation [12]. However, recent findings have demonstrated that its mechanism of action also involves interference with the Wnt/β-catenin, mTOR, and Jak/STAT signaling pathways, rendering it a promising candidate for drug repurposing [13]. Efforts towards the goal of discovering a novel clinical use of this drug have mainly focused on the treatment of cancer [14] and viral infections [15], without failing though to explore other diseases as well, such as inflammatory airway disease [16] and metabolic disorders [17].

In spite of extensive research, niclosamide’s potential as an anti-fibrotic agent is just starting to emerge, with findings supporting its potent anti-fibrotic effects against liver fibrosis [18], renal fibrosis [19], amyotrophic lateral sclerosis (ALS) [20], and graft-versus-host [21] related fibrotic events. Regarding the lung, we have previously identified niclosamide as a promising therapeutic agent for IPF in an in silico drug repurposing study using gene expression data from patients with various degrees of pulmonary fibrosis [22]. The promise of niclosamide as a possible anti-fibrotic agent has been verified by in vitro experiments on murine lung fibroblasts, a key player in the production of ECM, where treatment with niclosamide attenuated the activating effect of macrophage secreted S100a4 [23]. To further supplement the idea, Raju Boyapally et al. showed that in vivo treatment with niclosamide in an experimental bleomycin-induced IPF mouse model is capable of ameliorating lung fibrosis through epithelial to mesenchymal transition (EMT) inhibition [24].

Herein we aimed to investigate the suggested anti-fibrotic properties of niclosamide by employing an in vitro model of fibrosis using human primary subepithelial lung myofibroblasts (SELMs), and we have confirmed that this drug exerts a strong inhibitory effect on the fibrotic behavior of SELMS activated with pro-inflammatory and pro-fibrotic cytokines.

## 2. Materials and Methods

### 2.1. Patients

Lung tissue for myofibroblast isolation was obtained from four individual patients undergoing thoracic surgery for primary lung tumors with no evidence of ILD disease and no tumor presence in the respective tissue samples upon histopathological examination, which from now on are referred to as healthy controls. Healthy controls’ age and gender are listed in Table 1. All healthy controls were informed and gave their written consent prior to specimen collection. The study was approved by the local Research Ethics Committee of the University Hospital of Alexandroupolis (Protocol Number: 507/03-06-2019).

### 2.2. Chemicals

Niclosamide (Sigma-Aldrich, St. Louis, MI, USA) working solutions were prepared prior to each experiment and used immediately after preparation to avoid precipitation. More specifically, niclosamide was first weighed and diluted into a 1:1 solution of methanol:acetone (Sigma-Aldrich, St. Louis, MI, USA), followed by intense vortexing to yield a homogenous solution of 20 mM. The working solution was subsequently prepared through serial dilutions in dimethyl sulfoxide (DMSO; Sigma-Aldrich, St. Louis, MI, USA) to a concentration of 10 μM. Adequate quantities of the working solution were then diluted in a serum-free culture medium to yield the 30 nM and 100 nM desired concentrations for SELM stimulation. The final concentration percentage of DMSO in our cell cultures was 0.003% and 0.01% for the 30 nM and 100 nM concentrations of niclosamide, respectively. The same solvent used for niclosamide dilution was used as blank in SELM stimulations with no effect on SELM mRNA transcription, collagen production, or migration (Supplementary Table S1).

### 2.3. Subepithelial Lung Myofibroblast Isolation

Subepithelial lung myofibroblast (SELM) isolation and culture were performed as previously described [25]. Briefly, healthy lung tissue specimens were collected in Hank’s balanced salt solution (HBSS; Biosera, Cholet, France) with Ca^2+^, Mg^2+^, and antibiotics (penicillin 100 U/mL, streptomycin 100 mg/mL, amphotericin B 2.5 mg/mL and gentamicin 50 mg/mL; Biosera, Cholet, France). After 3 washes with HBSS with Ca^2+^, Mg^2+^ (HBSS+) and another 3 with HBSS without Ca^2+^, Mg^2+^ (HBSS-), tissue was de-epithelialized for 15 min in HBSS- containing 1 mM dithiothreitol (DTT) (Sigma-Aldrich, St. Louis, MI, USA) followed by 3 more incubations in HBSS- with ethylenediaminetetraacetic acid (EDTA) (Sigma-Aldrich, St. Louis, MI, USA) at 37 °C. Finally, tissue samples were placed in culture flasks containing Dulbecco’s modified Eagle’s medium (DMEM) (Biosera, Cholet, France) supplemented with 10% fetal bovine serum (FBS) (Biosera, Cholet, France) and antibiotics (penicillin 100 U/mL, streptomycin 100 mg/mL and amphotericin B 2.5 mg/mL) and incubated in 5% CO_2_ at 37 °C for up to 4 weeks. During this time, SELM colonies started to form, and when complete confluence was reached, SELMs were characterized by confirming the expression of a-smooth muscle actin (a-SMA, FITC conjugated, dilution 1:50; Abcam plc, Cambridge, UK) and vimentin (FITC conjugated, dilution 1:50; Abcam plc, Cambridge, UK) and the lack of expression of desmin (FITC conjugated, dilution 1:50; Abcam plc, Cambridge, UK) using a fluorescence microscope (Leica DM2000, Leica Microsystems GmbH, Wetzlar, Germany), as shown in Supplementary Figure S1.

### 2.4. Subepithelial Lung Myofibroblast Culture

SELMs were cultured in DMEM enriched with 10% FBS and antibiotics until 95% confluence before passaging. SELM cultures between passages 2 and 5 and at 95% confluence were used in experiments after a starvation period with DMEM containing antibiotics but not FBS for 24 h. Stimulations were performed with IL-1a (5 ng/mL; Novus biologicals, Littleton, CO, USA), TNF-a (50 ng/mL; Novus biologicals, Littleton, CO, USA), the combination of the two cytokines, IL-1a and TNF-a (2C; two cytokines), and TGF-β_1_ (5 ng/mL; Novus biologicals, Littleton, CO, USA) alone or in combination with niclosamide (Sigma-Aldrich, St. Louis, MI, USA) at 30 nM or 100 nM final concentration. The concentration of the aforementioned cytokines was chosen based on previous studies from our team [25,26,27], during which we observed that the in vitro pro-inflammatory and pro-fibrotic models were successfully established, while the niclosamide concentration was chosen based on the work of Boyapally et al. [24]. SELMs were incubated with cytokines with or without niclosamide at the aforementioned concentrations, and cell lysates for RNA and cell culture supernatants for collagen measurement were collected at 6 and 48 h, respectively. Again, the experimental time was chosen based on previous studies from our team [25,26,27], during which we observed that chosen experimental time window was appropriate for the in vitro pro-inflammatory and pro-fibrotic models.

### 2.5. Wound-Healing Assay

The migratory capability of SELMS was assessed by the performance of a scratch-wound assay as previously described [25]. Specifically, a micro-pipette tip was used to create a mechanical wound on SELMs cultured in 6-well plates and at 95% confluence. The migration of both unstimulated SELMs and SELMs stimulated with TGF-β_1_ (5 ng/mL), under the effect of 30 nM and 100 nM niclosamide, was then measured at time points 0 and 24 h after wound infliction. Photographs of the same area at different time points were taken with the help of pre-drawn lines vertical to the wound at the bottom of each well on an inverted Olympus (CKX53 LED) cell culture microscope (OLYMPUS EUROPA SE & CO. KG Hamburg, Germany). The percent migration of SELMs was quantified by analyzing the wound gap area closure using the ImageJ software (Fiji v.2.9.0; open source image processing package under the GNU General Public License) for scientific image analysis [28].

### 2.6. Collagen Production

Production of secreted collagen by SELMs was measured with the use of a commercially available Sircol collagen assay (Biocolor, Carrickfergus, UK) according to the manufacturer’s instructions. In brief, 200 μL of ice-cold collagen concentration and isolation reagent were added to 1 mL of cell culture supernatant from each experimental condition and incubated overnight in ice. Samples were then centrifuged, and 1 mL of Sircol dye reagent per sample was added, followed by a 30-min incubation on a mechanical shaker. After centrifugation, the visible collagen pellet was washed with 750 μL of ice-cold acid-salt wash reagent, and the collagen-bound dye was then released by the addition of 250 μL alkali reagent. The ODs of samples and reaction standards were measured in a microplate reader (Diareader EL×800; Dialab, Wr. Neudorf, Austria) at 540 nm against the OD of fresh culture medium as a blank. Collagen concentration was calculated using the linear curve generated by the ODs of the reaction standards.

### 2.7. Immunofluorescence

For immunofluorescence staining, SELMs were cultured on 8-well chamber slides (NUNC, Roskilde, Denmark) until 95% confluence and subsequently fixed with ice-cold 4% paraformaldehyde (PFA; Sigma-Aldrich, St. Louis, MI, USA) for 30 min. Blocking for nonspecific staining was performed by 60-min incubation with a 5% bovine serum albumin (BSA; Sigma-Aldrich, St. Louis, MI, USA) solution in PBS (Sigma-Aldrich, St. Louis, MI, USA) before staining for α-SMA (Abcam plc, Cambridge, UK) and CD90 (Novus biologicals, Littleton, CO, USA) at 1:50 and 1:200 dilutions, respectively. A secondary antibody (Goat anti-rabbit IgG, conjugated with FITC; Merck Millipore, Burlington, MA, USA) was then added to the slides at a 1:100 dilution, and nuclei were stained with DAPI (Sigma-Aldrich, St. Louis, MI, USA) before being studied with a fluorescence microscope (Leica DM2000, Leica Microsystems GmbH, Wetzlar, Germany).

### 2.8. Human Fibronectin Enzyme-Linked Immunosorbent Assay

Human fibronectin was measured in SELM culture supernatants using a commercially available kit (human fibronectin enzyme-linked immunosorbent assay [ELISA] kit, Origene, Rockville, MD, USA) as previously described [27] and according to the manufacturer’s instructions. In brief, SELM supernatants and fibronectin standard solutions were added to a pre-coated 96-well plate before adding a biotinylated anti-human fibronectin antibody. ABC working solution was added to each well, and TMB color developing agent was later added to the samples and standards. The reaction was stopped by the addition of TMB stop solution, and the optical density absorbance was measured at 450 nm on a microplate reader (Diareader EL×800; Dialab, Wr. Neudorf, Austria). The concentration of fibronectin was calculated using a standard curve according to the manufacturer’s instructions.

### 2.9. Caspase-3 Activity Assay

The catalytic activity of caspase-3 was measured using the caspase-3 colorimetric assay kit (Merck Millipore, Burlington, MA, USA) according to the manufacturer’s instructions. In brief, SELMs cultured with the combination of 2 cytokines and TGF-β_1_, with or without niclosamide at 100 nM concentration, were collected in 200 μL chilled cell lysis buffer and incubated on ice for 10 min. Following centrifugation at 10,000× *g* for 10 min, 70 μL of the cell supernatants were transferred to a new tube containing 20 μL of 5× assay buffer and 10 μL of the caspase-3 substrate Ac-DEVD-pNA and incubated for 2 h at 37 °C. At the end of the incubation period, 100 μL of each sample was transferred to a 96-well plate, and their absorbance at 405 nm was measured on a microplate reader (Diareader EL×800; Dialab, Wr. Neudorf, Austria). The concentration of the pNA product was calculated from the absorbance of a standard curve of known concentrations.

### 2.10. Total RNA Isolation and Purification

Total RNA was isolated using the NucleoZol reagent (MACHEREY-NAGEL GmbH & Co, Dueren, Germany) according to the manufacturer’s instructions. More specifically, 0.15 × 10^6^ seeded SELMs that were cultured in 6-well plates were lysed by the addition of 500 μL of NuceloZol reagent per well and thorough pipetting. Samples were transferred to tubes, and 200 μL of RNase-free water was added to each sample. Samples were centrifuged for 15 min at 12,000× *g* to precipitate contaminants, and 500 μL of supernatant was transferred to a new tube, followed by the addition of 500 μL of isopropanol for RNA precipitation. Finally, RNA was washed twice with 75% ethanol, reconstituted in RNase-free water, and measured using the UV-Vis Spectrophotometer Q5000 (Quawell, San Jose, CA, USA) for concentration and purity determination. To eliminate possible DNA contamination, RNA samples were treated with deoxyribonuclease I (recombinant DNase I, RNase-free; TAKARA, Kusatsu, Shiga, Japan) for 15 min followed by DNase inactivation with heat and EDTA (Sigma-Aldrich, St. Louis, MI, USA).

### 2.11. cDNA Synthesis and Real-Time RT-PCR

cDNA synthesis and real-time PCR were performed as previously described [27]. Briefly, cDNA was synthesized from 250 ng RNA with the use of PrimeScript RT reagent kit (Perfect Real Time; TAKARA, Kusatsu, Shiga, Japan), and 10 μL reactions for quantitative real-time (qRT)-PCR was prepared using the 2xKAPA SYBR FAST qPCR Kit (Kapa Biosystems Ltd., Boston, MA, USA) containing 25 ng of cDNA, 5 μL of 2xKAPA SYBR FAST reaction Mastermix and 200 nM of the forward and reverse primers of each gene (Table 2). Gene amplification was performed in a SaCycler-96 real-time PCR system (Sacace Biotechnologies, Como, Italy) at 60 °C annealing temperature using a two-step cycling protocol. At the end of each reaction, a melting curve was calculated from 45 °C to 95 °C, counting fluorescence in 1 °C increments. The expression of the targeted genes was normalized against the expression of the housekeeping gene *GAPDH* in the same sample using the 2^−ΔΔCt^ method [29].

**Table 2:** Forward and reverse primer sequences per gene for RT-PCR.

Gene	Forward Primer	Reverse Primer	Reference
*GAPDH*	GACATCAAGAAGGTGGTGAA	TGTCATACCAGGAAATGAGC	[27]
Collagen Type I (*COL1*)	CCCTGGAAAGAATGGAGATGAT	ACTGAAACCTCTGTGTCCCTTCA	
Collagen Type III (*COL3*)	GCTCTGCTTCATCCCACTATTA	TGCGAGTCCTCCTACTGCTAC	
Fibronectin (*FN*)	CCAGTCCACAGCTATTCCTG	ACAACCACGGATGAGCTG	
α-sma (*ACTA-2*)	AATGCAGAAGGAGATCACGG	TCCTGTTTGCTGATCCACATC	
*CD90*	CGCTCTCCTGCTAACAGTCTT	CAGGCTGAACTCGTACTGGA	[30]

### 2.12. Statistics

The mean values of the data with standard deviations (SD) are shown in Section 3. Statistical comparison between the groups was performed using one-way ANOVA after testing the data for normality using the Kolmogorov–Smirnov test, and statistical significance was established as a *p*-value < 0.05.

## 3. Results

### 3.1. Niclosamide Treatment Attenuates SELM Fibrotic mRNA Expression in Response to Inflammatory Stimuli

**Figure 1:**
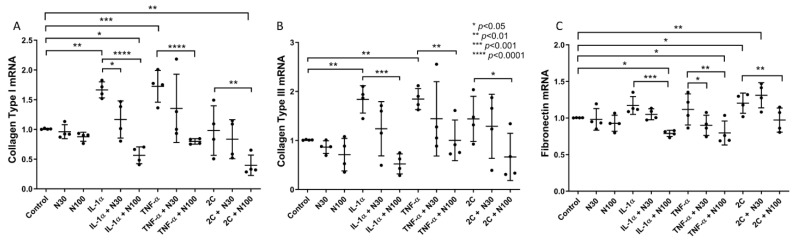
The effect of niclosamide on the IL-1α and TNF-α-inducible mRNA expression of *collagen type I*, *collagen type III*, and *fibronectin*. *Collagen type I* (**A**), *collagen type III* (**B**), and *fibronectin* (**C**) mRNA expression levels in SELMs after stimulation with IL-1α, TNF-α or their combination, treated or not with niclosamide at 30 nM and 100 nM concentrations. Mean values of data from experiments performed in triplicates on SELMs from 4 individuals are shown. 2C: IL-1α + TNF-α, N30: niclosamide 30 nM, N100: niclosamide 100 nM.

To investigate niclosamide’s anti-fibrotic effect in vitro, we treated SELMs with two different concentrations of niclosamide (NCL), namely 30 nM (N30) and 100 nM (N100), after stimulation with pro-inflammatory cytokines. Overall, treatment of stimulated SELMs with niclosamide led to a reduction of *collagen type I* (*COL1*), *collagen type III* (*COL3*), and *fibronectin* (*FN*) mRNA transcription in a concentration-dependent manner (Figure 1).

More specifically, stimulation of SELMs with the pro-inflammatory cytokines TNF-α and IL-1α upregulated the baseline mRNA expression of *COL1* (IL-1α: 1.66-fold, ±0.13, *p* < 0.01; TNF-α: 1.72-fold, ±0.26, *p* < 0.001) (Figure 1A) and *COL3* (IL-1α: 1.83-fold, ±0.27, *p* < 0.01; TNF-α: 1.84-fold, ±0.21, *p* < 0.01) (Figure 1B) compared to unstimulated SELMs, as expected from previous bibliography reports [27]. Stimulation with both TNF-α and IL-1α (2C) resulted in upregulated mRNA expression of *FN* (2C: 1.2-fold, ±0.13, *p* < 0.05) (Figure 1C) as well as *COL1* and *COL3*, though it failed to reach statistical significance.

Treatment with NCL alone did not have a statistically significant effect on the mRNA expression of *COL1*, *COL3*, or *FN* (Figure 1A–C). However, *COL1* mRNA production by SELMs in response to either IL-1α (IL-1α + N30: 1.16-fold, ±0.31, *p* < 0.05; IL-1α + N100: 0.53-fold, ±0.14, *p* < 0.0001), TNF-α (TNF-α + N100: 0.79-fold, ±0.04, *p* < 0.0001), or their combination (2C + N100: 0.39-fold, ±0.17, *p* < 0.01) was reduced after NCL treatment, with the effect of the higher concentration (N100) being more pronounced. Notably, the reduction of *COL1* mRNA expression after N100 treatment of IL-1α and 2C stimulated SELMs was statistically significant even when compared to controls (IL-1α + N100: *p* < 0.05; 2C + N100: *p* < 0.01) (Figure 1A).

A similar pattern was observed with the mRNA expression levels of *COL3* where the addition of N100, after stimulation with ΙL-1α (IL-1α + N100: 0.52-fold, ±0.2, *p* < 0.001), TNF-α (TNF-α + N100: 1.0-fold, ±0.41, *p* < 0.01) and 2C (2C + N100: 0.66-fold, ±0.48, *p* < 0.05) effectively counteracted the effect of the pro-inflammatory cytokines (Figure 1B).

Finally, *FN* mRNA expression was shown to be less inducible by pro-inflammatory cytokines; however, NCL treatment attenuated the effect of IL-1α (IL-1α + N100: 0.78-fold, ±0.04, *p* < 0.001), TNF-α (TNF-α + N30: 0.9-fold, ±0.13, *p* < 0.05; TNF-α + N100: 0.79-fold, ±0.016, *p* < 0.01), and 2C (2C + N100: 0.97-fold, ±0.16, *p* < 0.01), with N100 proving to be most effective, and its effect on *FN* mRNA expression was even observable when compared to unstimulated SELMs (IL-1α + N100: *p* < 0.05; TNF-α + N100: *p* < 0.05). In contrast, when SELMs were treated with N30, following stimulation with 2C, *FN* mRNA expression was induced (2C + N30: 1.31-fold, ±0.17, *p* < 0.01) (Figure 1C).

Furthermore, in order to test whether the niclosamide-attenuated collagen mRNA expression of SELMs was translated into decreased protein collagen secretion as well, we proceeded to measure the total collagen secreted by cells in their culture medium after treatment with NCL. After NCL treatment of SELMs, stimulated with pro-inflammatory cytokines, there was an observable slight decrease in protein collagen production which proved to be inconsistent and not statically significant (Supplementary Table S2).

### 3.2. Niclosamide Treatment Attenuates SELM Fibrotic Expression in Response to Fibrotic Stimuli

As pro-inflammatory cytokine stimulation did not induce the protein expression of collagen (Supplementary Table S2), we proceeded with studying the niclosamide’s anti-fibrotic effect using the well-established and well-studied TGF-β fibrosis in vitro model [25,26,27,31]. In addition, having observed that NCL at 100 nM concentration exhibited the most promising inhibitory potency on SELM fibrotic response, we chose to proceed with using only this concentration for subsequent treatments.

**Figure 2:**
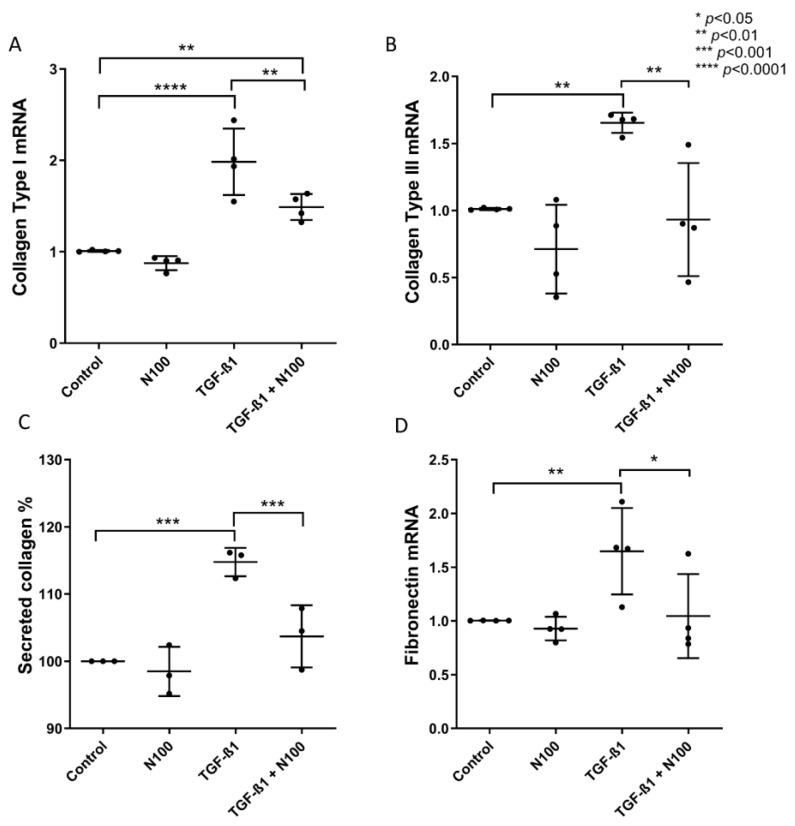
The effect of niclosamide on the TGF-β_1_-inducible expression of *collagen type I*, *collagen type III*, and *fibronectin*. *Collagen type I* (**A**), *collagen type III* (**B**), and *fibronectin* (**D**) mRNA expression levels in SELMs after stimulation with TGF-β_1_ with or without treatment with niclosamide at 100 nM concentration. Total secreted collagen from SELMs stimulated with TGF-β_1_ with or without treatment with niclosamide at 100 nM concentration (**C**). Mean values of data from experiments performed in triplicate on SELMs from 4 individuals (**A**,**B**,**D**) and 3 individuals (**C**) are shown. N100: niclosamide 100 nM.

Upon stimulation with TGF-β_1_, mRNA expression levels of *COL1* (1.98-fold, ±0.36, *p* < 0.0001) (Figure 2A), *COL3* (1.65-fold, ±0.07, *p* < 0.01) (Figure 2B), as well as *FN* (1.64-fold, ±0.40, *p* < 0.01) (Figure 2D) were upregulated. The addition of N100 to TGF-β_1_-stimulated SELMs led to a significant reduction in the expression of *COL1* (TGF-β_1_ + N100: 1.48-fold, ±0.14, *p* < 0.01), *COL3* (TGF-β_1_ + N100: 0.93-fold, ±0.42, *p* < 0.01), and *FN* (1.04-fold, ±0.39, *p* < 0.05) (Figure 2D). Interestingly, even though *COL1* mRNA expression after TGF-β_1_ stimulation was reduced in N100-treated SELMs, it still exhibited upregulation when compared to controls (TGF-β_1_ + N100: *p* < 0.01) (Figure 2A).

The mRNA expression of *α-SMA* was also induced by TGF-β_1_ (1.40-fold, ±0.22, *p* < 0.01) (Supplementary Figure S2A) in contrast to *CD90* which demonstrated no significant difference between unstimulated and TGF-β_1_ stimulated cells (Supplementary Figure S2B). Notably, the addition of N100 led to the reduction of *α-SMA* expression levels in response to TGF-β_1_ (0.99-fold, ±0.12, *p* < 0.01) (Supplementary Figure S2A), while *CD90* expression was diminished below baseline (0.57-fold, ±0.13), which translated to a significant reduction in mRNA expression when compared to both unstimulated (*p* < 0.01) and TGF-β_1_-stimulated SELMs (*p* < 0.05) (Supplementary Figure S2B).

At a protein level, total collagen production of SELMs was upregulated in response to TGF-β_1_ stimulation (114.8%, ±2.11, *p* < 0.001), and after a 48-h treatment with N100, it was indeed reduced to baseline levels (103.7%, ±4.62, *p* < 0.001) (Figure 2C). SELMs stained for α-SMA (Supplementary Figure S3A) and CD90 (Supplementary Figure S3B) showed no difference in the expression of these proteins between unstimulated and TGF-β_1_-stimulated cells, and there was no observable effect of NCL treatment. Regarding protein production of fibronectin by SELMs, neither TGF-β_1_ nor NLC alone or in combination with TGF-β_1_ had any significant effect on secreted fibronectin levels, suggesting maybe that NCL majorly affects collagen production, a main ECM component, and not secondary components, such as fibronectin.

### 3.3. Niclosamide Inhibits SELM Migration

Migration of activated myofibroblasts across a wound gap plays a major role in both the physiological and pathological processes of wound healing and fibrosis, respectively. As we have previously demonstrated that treatment of SELMs with TGF-β_1_ stimulates their migration [25], we, therefore, investigated their migratory capability after the infliction of a mechanical wound in the presence of TGF-β_1_ and whether it could be altered by the addition of niclosamide at the higher concentration used in our experiments.

**Figure 3:**
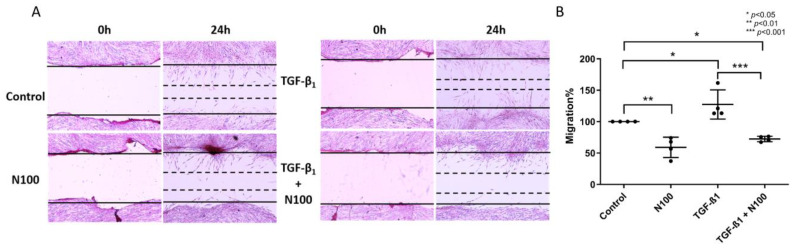
The effect of niclosamide on the TGF-β1-inducible migration of SELMs. (**A**) Scratch wound assay on SELMs cultured in 6-well plates and stained with H&E from representative wells at 40× magnification. SELM migration after TGF-β_1_ stimulation, with or without treatment with niclosamide at 100 nM concentration, is estimated by the percentage of wound closure at 24 h. (**B**) Dot plot representation of SELM migration after TGF-β_1_ stimulation, with or without treatment with niclosamide at 100 nM concentration calculated from the scratch wound assay using ImageJ. Mean values of data from experiments performed in triplicate on SELMs from 4 individuals are shown. Magnification 40×; N100: niclosamide 100 nM.

As shown in Figure 3, at 24 h after wound infliction, SELMs had formed a visible front of cells moving towards each other, closing the gap of the wound. This behavior was enhanced by stimulation with TGF-β_1_, where considerably more gap closure could be observed at the 24-h time point (127.3%, ±23.17, *p* < 0.05) (Figure 3B). N100 exposure hindered the ability of SELMs to migrate even at standard conditions (59.04%, ±16.15, *p* < 0.01). The debilitating effect of NCL on SELM migration was even more prominent when combined with TGF-β_1_ stimulation (72.44%, ±4.33), leading to a 41% reduction in migration compared to controls (*p* < 0.05) and a 43% reduction when compared to TGF-β_1_ stimulation alone (*p* < 0.001) (Figure 3A,B).

Finally, in order to test whether the inhibition of SELM protein collagen production and migration observed after treatment with niclosamide is dependent on the induction of apoptosis, we continued to measure the catalytic activity of caspase-3. After 48 h of incubation, there was no observable apoptotic activity in unstimulated cells, and the same effect was true for SELMs stimulated with either the two cytokines (2C) or TGF-β1. In addition, treatment with N100 alone or in combination with 2C or TGF-β1 did not lead to an induction of caspase-3 activity compared to either unstimulated or stimulated cells (Supplementary Table S3).

## 4. Discussion

In this study, we examined the role of niclosamide (NCL), a known anti-parasitic drug, as a novel anti-fibrotic agent for interstitial lung disease (ILD) and especially idiopathic pulmonary fibrosis (IPF). Our results demonstrate that this compound not only strongly attenuates the mRNA and protein expression of collagen types I and III, as well as fibronectin, in primary human subepithelial lung myofibroblasts (SELMs) but also inhibits their migration under the pro-fibrotic conditions mimicking ILD.

Our results confirm that niclosamide has strong effects on the physiology of human myofibroblasts, ablating their pro-fibrotic behavior under inflammatory and fibrotic stimuli in a concentration-depended manner. After initially proposing NCL as a potential anti-fibrotic agent in an in silico study exploring the genetic signature of IPF for drug repurposing [22], we have now verified this finding in an in vitro model of fibrosis. Although the anti-fibrotic activity of niclosamide has been studied before in an in vivo murine model of bleomycin-induced pulmonary fibrosis [24], there are some well-known disadvantages that are intrinsic to the use of animals as well as to the specific model, that restrict the translatability of the results [32]. The results of our study in primary human lung myofibroblasts provide further evidence of the anti-fibrotic potency of niclosamide in humans and support the idea of repurposing this drug for IPF.

From what is already known, IPF pathogenesis is primarily composed of a fibrogenic component involving the production of extracellular matrix by fibroblasts and myofibroblasts [33], with inflammation playing a less important but nonetheless defined role in its initiation and progression [34]. Tissue fibrosis is considered the end result of repetitive alveolar damage leading to chronic inflammation [34]. Innate immune system activation and attraction of neutrophils and macrophages can activate lung mesenchymal cells to produce ECM via the secretion of pro-inflammatory and pro-fibrotic mediators, such as TNF-α, IL-1α, INF-γ, TGF-β_1_, and others [35]. Upon activation by TGF-β_1_, fibroblasts differentiate into myofibroblasts which are characterized by the aberrant synthesis of collagen driving pathologic ECM deposition and remodeling [33]. Collagen types I and III, as well as fibronectin, are primarily produced by activated myofibroblast during normal wound healing responses [36] and are major components of abnormal ECM accumulations found in IPF and other fibrotic conditions [37].

As we demonstrated in this study, treating activated myofibroblasts with niclosamide at two different concentrations resulted in dampened mRNA expression of *collagen types I*, *III*, and *fibronectin*, as well as in decreased collagen production at the protein level in response to pro-fibrotic stimuli. This result could be attributed to niclosamide’s pharmacologic activity to act as an inhibitor of both TGF-β_1_ and the canonical Wnt signaling pathways (8). Activation of Wnt and subsequent phosphorylation of β-catenin is known to promote fibrosis in various organs, acting synergistically with TGF-β_1_ signaling [38]. Additionally, the Wnt/β-catenin pathway has been found to be overexpressed in alveolar epithelial type II (ATII) cells and other cells in IPF-afflicted pulmonary tissue [39], thus supporting the hypothesis that niclosamide could ameliorate disease manifestations through inhibition of this pathway. In this study, we show a strong inhibitory effect of niclosamide on primary ECM components expression from SELMs. Our results agree with the ones of Raju Boyapally et al., which confirm niclosamide’s anti-fibrotic activity in vivo through inhibition of TGF-β_1_ and Wnt/β-catenin pathways [24]. Further than that, additional evidence that niclosamide could indeed be used as an anti-fibrotic drug comes from studies in animal models of the liver [18,40] and renal fibrosis [19], where treatment with niclosamide was able to attenuate the fibrotic effects of various manipulations.

Niclosamide’s multimodal activity against a variety of cellular targets and processes renders it exceedingly challenging to pinpoint the exact mechanism of action that is responsible for the desired pharmacological effect [13]. Apart from TGF-β_1_ and the Wnt/β-catenin pathway, niclosamide has also been demonstrated to function through oxidative phosphorylation disruption in the mitochondria, which seems to be of particular importance for its anti-proliferative properties in cancer cells [41,42], and has not been yet comprehensibly studied regarding its involvement in fibrosis amelioration. Thus, further studies are needed in order to elucidate the extent to which the mechanism contributes to the anti-fibrotic effects of niclosamide.

Although the mRNA expression of *collagen type I*, *collagen type III*, and *fibronectin* was upregulated in response to pro-inflammatory stimuli, and niclosamide treatment led to significant inhibition of this effect, this result was not reproduced when examining total collagen production at a protein level. This result is in agreement with previous findings, reporting minimal [26] or no effect [25] of pro-inflammatory cytokine stimulation on the pro-fibrotic behavior of myofibroblasts. On the other hand, it is already known that TGF-β induces fibrotic responses in myofibroblasts, and we and others have proven that this constitutes an excellent in vitro model for studying fibrosis [25,26,27,31,43,44,45]. Therefore, we proceeded to study niclosamide’s effect on TGF-β-induced collagen expression and found that at both mRNA and protein levels, niclosamide exerted an inhibitory effect. Similar results were observed during the cell migration experiments, where niclosamide abolished the TGF-β-mediated induction of SELM migration. Interestingly, the same pattern was observed with the *α-SMA* and *CD90* mRNA expression, which, nonetheless, was not confirmed at the protein level, indicating that niclosamide’s inhibitory effect is mainly focused on SELM migration and collagen production rather than other ECM components.

Furthermore, we demonstrated a robust restriction of myofibroblast migration, both unstimulated and in response to TGF-β_1_, after the addition of niclosamide at high concentrations. Under physiological conditions, myofibroblast contractile activity and migration, together with ECM production, are essential for a proper wound-healing response and scar tissue formation [46]. However, in the context of fibrotic diseases, aberrant activation of myofibroblasts with excessive cell motility and contraction force production leads to ECM re-organization, increasing stiffness and causing loss of function in the affected tissue [47,48]. Mitigating the effects of myofibroblast contractility and migratory activity is, therefore, critical for the restoration of the physiological properties of ECM and, ultimately, the recovery of organ function, and to this end, niclosamide’s anti-migratory potential may offer some therapeutic benefit to fibrotic diseases such as IPF.

Apoptosis of activated myofibroblasts is an important hallmark of normal wound healing, and apoptotic-resistant myofibroblasts have been reported to play a key role in the development of IPF [49]. Initiation of this process by both intrinsic and extrinsic pathways leads to the cleavage and subsequent activation of caspase-3, resulting in programmed cell death [50]. Niclosamide is known to induce apoptosis in various cancer cell lines; however, it was less likely to exhibit the same effect on normal esophageal epithelial cells and fibroblasts [51]. From our experiments, we did not observe a measurable induction of apoptosis in SELMs treated with 100 nM of niclosamide. Nevertheless, it is reported that niclosamide incubation of fibroblast-like synoviocytes from patients with rheumatoid arthritis was able to induce apoptosis and increase the levels of cleaved caspase-3, though this was observed at concentrations higher than 250 nM [52]. Further than that, another study has reported that the addition of niclosamide at concentrations lower than 1 μM was found to have minimal effects on fibroblast viability [53], possibly indicating that niclosamide’s pro-apoptotic activity on healthy fibroblasts becomes important at higher concentrations.

Current options for IPF pharmacotherapy are still limited, with the most prominent being the two newly approved compounds, pirfenidone and nintedanib [54]. Even though these two compounds have been observed to delay the development of the disease and worsening of symptoms, they are unable to stop or reverse the progression of IPF [55], stressing the need for discovering more efficacious treatments. Although not entirely understood, pirfenidone’s mode of action mainly involves inhibition of the TGF-β_1_/Smad3 signaling pathway [56,57], whereas nintedanib is primarily a tyrosine kinase inhibitor of multiple growth factor receptors such as vascular endothelial growth factor (VEGF), platelet-derived growth factor (PDGF) and fibroblast growth factor (FGF) [58]. Niclosamide’s pleiotropic pharmacological activity and distinct mode of action through Wnt pathway inhibition [13] could therefore supplement the anti-fibrotic activity of these drugs and even increase their efficacy.

In conclusion, with this study, we show that niclosamide treatment has a strong inhibitory effect on primary human lung myofibroblasts’ collagen and fibronectin expression. Total secreted collagen was also decreased when assessed at the protein level. After niclosamide treatment, the myofibroblast migration rate was also diminished even below baseline levels. Altogether, these results provide evidence that niclosamide could hold promise as a novel anti-fibrotic therapy for interstitial lung diseases such as IPF.

## Figures and Tables

**Table 1:** Characteristics of healthy controls that were included in the study.

Identifier	Age	Gender
1	59	Male
2	51	Female
3	64	Male
4	70	Male

## Data Availability

No new data were created during this study.

## Supplementary Materials

The following supporting information can be downloaded at: https://www.mdpi.com/article/10.3390/biomedicines11072032/s1, Figure S1: Characterization of SELMs through immunofluorescence staining for α-SMA, vimentin, and desmin. SELMs are characterized by the expression of α-SMA and vimentin and the absence of desmin expression. Cell nuclei are stained with DAPI; Figure S2: The mRNA and protein expression of α-SMA and CD90 after treatment with TGF-β_1_ and niclosamide. α-SMA (A) and CD90 (B) mRNA expression after treatment with niclosamide at 100 nM concentration, TGF-β_1_ at 5 ng/mL, or their combination. Mean values of data from experiments performed in triplicate on SELMs from 4 individuals are shown. N100: niclosamide 100 nM; Figure S3: Immunofluorescence for α-SMA (A) and CD90 (B) treated with niclosamide at 100 nM concentration, TGF-β_1_ at 5 ng/mL, or their combination. Nuclei are stained blue with DAPI, and α-SMA and CD90 are stained green. Magnification 400×; N100: niclosamide 100 nM; Table S1: The effect of 0.01% DMSO solvent on SELM mRNA transcription, collagen production, or migration; Table S2: The effect of Niclosamide on SELM collagen production; Table S3: The effect of Niclosamide on SELM caspase-3 activity.
