# Supplementary material for: Niclosamide Attenuates Inflammation-Associated Profibrotic Responses in Human Subepithelial Lung Myofibroblasts
Source: Biomedicines. 2023 Jul 19;11(7):2032. doi: 10.3390/biomedicines11072032 (PMC10377180; doi:10.3390/biomedicines11072032)
Supplement: Supplementary file 1 [file biomedicines-11-02032-s001.zip › biomedicines-2491312-supplementary.pdf]

Supplementary Materials

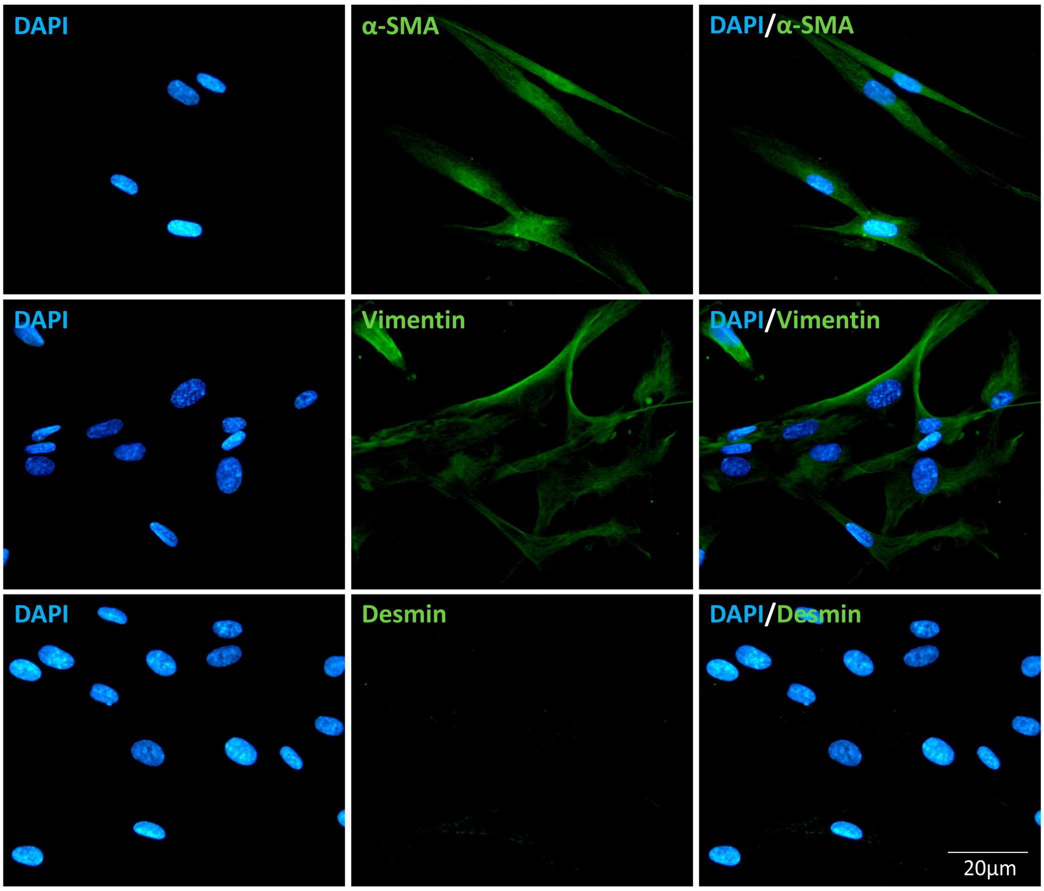

Figure S1. Characterization of SELMs through immunofluorescence staining for α-SMA, vimentin, and desmin. SELMs are characterized by the expression of α-SMA and vimentin and the absence of desmin expression. Cell nuclei are stained with DAPI.

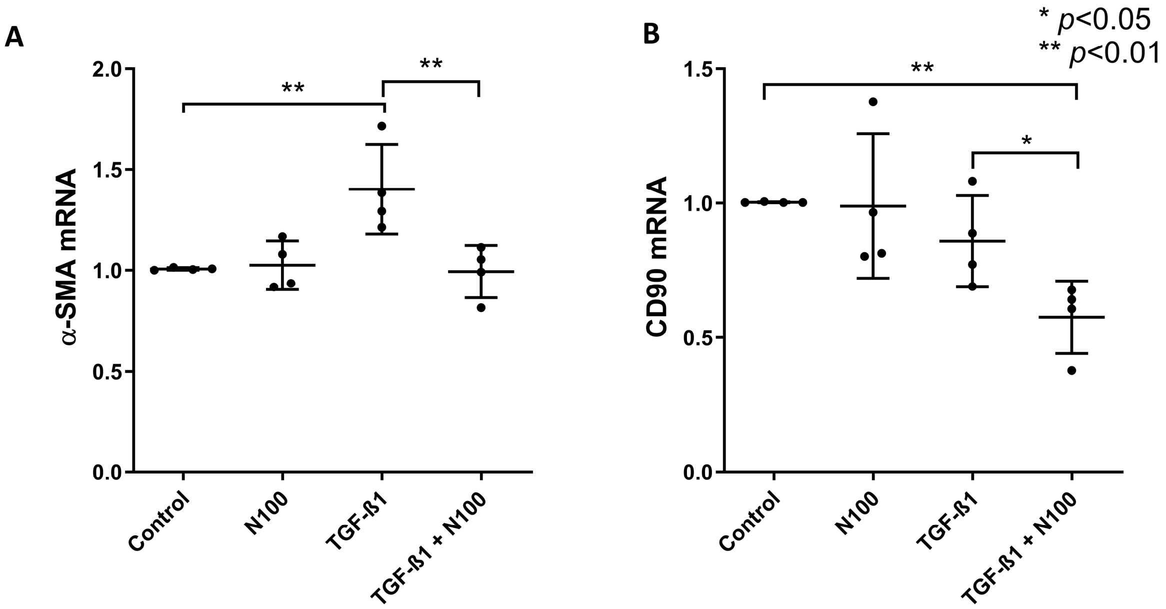

Figure S2. The mRNA and protein expression of α-SMA and CD90 after treatment with TGF-β1 and niclosamide. α-SMA (**A**) and CD90 (**B**) mRNA expression after treatment with niclosamide at 100 nM concentration, TGF-β1 at 5 ng/mL, or their combination. Mean values of data from experiments performed in triplicate on SELMs from 4 individuals are shown. N100: niclosamide 100 nM.

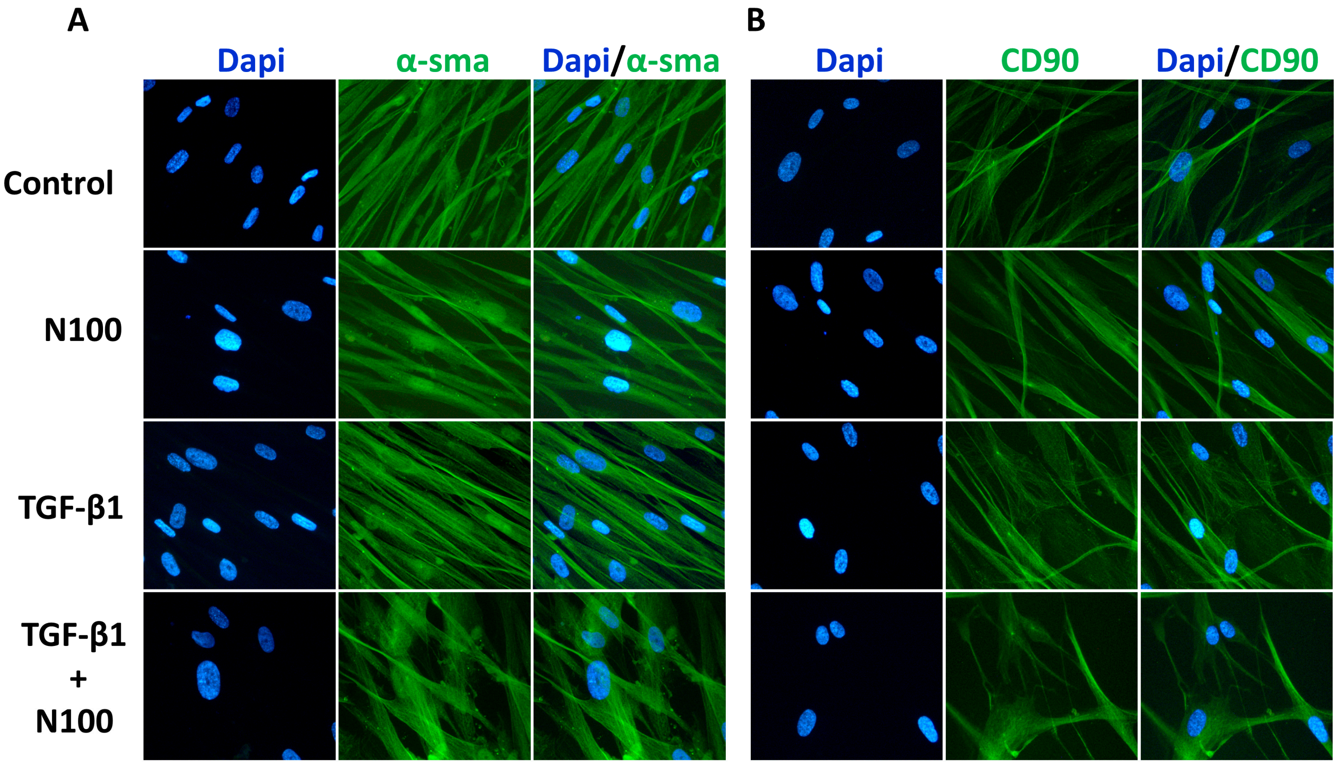

Figure S3. Immunofluorescence for α-SMA (**A**) and CD90 (**B**) treated with niclosamide at 100 nM concentration, TGF-β1 at 5 ng/mL, or their combination. Nuclei are stained blue with DAPI, and α-SMA and CD90 are stained green. Magnification 400×; N100: niclosamide 100 nM.

Table S1. The effect of 0.01% DMSO solvent on SELM mRNA transcription, collagen production, or migration.

Assay	Control (Unstimulated)	DMSO 0.01%	Statistical Significance
COL1 mRNA	1.01-fold ±0.05	1.11-fold ±0.02	None
COL3 mRNA	1.05-fold ±0.02	0.98-fold ±0.05	
FN mRNA	1.07-fold ±0.01	1.02-fold ±0.04	
ACTA-2 mRNA	0.99-fold ±0.02	1.01-fold ±0.07	
CD90 mRNA	1.11-fold ±0.04	0.97-fold ±0.02	
Collagen protein (Sircol)	102.4% ±5.39	100.7% ±1.81	
Migration	100.4% ±16.96	97.92% ±12.88	

Table S2. The effect of Niclosamide on SELM collagen production.

Assay	Control (Unstimulated)	N100	IL-1α+TNF-α	IL-1α+TNF-α+N100	Statistical Significance
Collagen protein (Sircol)	100.2% ±14.53	84.47-fold ±2.33	117.1-fold ±18.32	94.43-fold ±3.64	None

Table S3. The effect of Niclosamide on SELM caspase-3 activity.

Assay	Control (Unstimulated)	N100	IL-1α+TNF-α	IL-1α+TNF-α+N100	TGF-β	TGF-β+N100	Statistical Significance
Caspase-3 activity	Non detectable	Non detectable	Non detectable	Non detectable	Non detectable	Non detectable	None
